# Infodemics and infodemiology: a short history, a long future

**DOI:** 10.26633/RPSP.2021.40

**Published:** 2021-05-12

**Authors:** Chris Zielinski

**Affiliations:** 1 University of Winchester Winchester United Kingdom University of Winchester, Winchester, United Kingdom

**Keywords:** Coronavirus infections, SARS virus, health communication, pandemics, health information management, Infecciones por coronavirus, virus del SRAS, comunicación en salud, pandemias, gestión de la información en salud, Infecções por coronavirus, vírus da SARS, comunicação em saúde, pandemias, gestão da informação em saúde

## Abstract

An “infodemic” is defined as “an overabundance of information – some accurate and some not – occurring during an epidemic”. This paper describes the characteristics of an infodemic, which combines an inordinately high volume of information (leading to problems relating to locating the information, storage capacity, ensuring quality, visibility and validity) and rapid output (making it hard to assess its value, manage the gatekeeping process, apply results, track its history, and leading to a waste of effort). This is bound up with the collateral growth of misinformation, disinformation and malinformation. Solutions to the problems posed by an infodemic will be sought in improved technology and changed social and regulatory frameworks. One solution could be a new trusted top-level domain for health information. The World Health Organization has so far made two unsuccessful attempts to create such a domain, but it is suggested this could be attempted again, in the light of the COVID-19 infodemic experience. The vital role of reliable information in public health should also be explicitly recognized in the Sustainable Development Goals, with explicit targets. All countries should develop knowledge preparedness plans for future emergencies.

## RESUMEN

Una *infodemia* se define como ‘una sobreabundancia de información —que puede ser correcta o no— durante una epidemia’. En este artículo se describen las características de una infodemia, en la cual se combina un volumen de información desmesuradamente alto (que genera problemas que guardan relación con la búsqueda, la capacidad de almacenamiento, la calidad, la visibilidad y la validez de la información) y la producción acelerada de información (que hace difícil estimar su valor, gestionar el proceso de control, aplicar resultados y rastrear el historial, y además conduce al desperdicio de esfuerzos). Esto está vinculado con el crecimiento colateral de información errónea, la desinformación y la información malintencionada. Se exploran soluciones para los problemas ocasionados por una infodemia mediante tecnologías más avanzadas y cambios en los marcos sociales y regulatorios. Una solución podría ser un dominio de nivel superior nuevo y fidedigno para la información en materia de salud. Hasta el presente, la Organización Mundial de la Salud ha llevado a cabo dos intentos infructuosos de crear dicho dominio, pero se recomienda volver a intentarlo, considerando la experiencia con la infodemia de la COVID-19. Además, el papel clave que desempeña la información fiable en la salud pública debe reconocerse explícitamente en los Objetivos de Desarrollo Sostenible, estableciendo metas explícitas. Todos los países deben elaborar planes de preparación para la gestión del conocimiento con miras a emergencias futuras.

## RESUMO

Infodemia é definida como “um excesso de informações – algumas exatas e outras não – que ocorre em uma epidemia”. Este trabalho descreve as características de uma infodemia, que combina um volume extraordinariamente grande de informação (levando a problemas relacionados à localização, capacidade de armazenamento e garantia da qualidade, visibilidade e validade da informação) com produção acelerada (o que dificulta avaliar seu valor, gerenciar o processo de seleção de informação, aplicar resultados e rastrear seu histórico, resultando em um esforço em vão). Este fenômeno está atrelado ao crescimento colateral de informações falsas, desinformação e desinformação maliciosa. A busca de soluções aos problemas decorrentes de uma infodemia deve estar no aprimoramento da tecnologia e na modificação das estruturas regulatória e social. Uma solução seria criar um domínio de nível superior com credibilidade para informação em saúde. A Organização Mundial da Saúde (OMS) fez até o presente duas tentativas infrutíferas para criar tal domínio. Porém, se recomenda que uma nova tentativa seja feita em vista da experiência adquirida com a infodemia de COVID-19. O papel vital da informação confiável em saúde pública também deve ser expressamente reconhecido nos Objetivos de Desenvolvimento Sustentável, com metas explícitas. Todos os países devem elaborar planos de preparação em conhecimento para futuras emergências.

With the arrival of the COVID-19 pandemic, the output of information about this new global health threat began to grow at an exponential rate. According to Evidence Aid Chairman Professor Mike Clarke “Since the pandemic broke, over 75 000 scientific papers have been published on COVID-19 across the world and a new one is appearing every three minutes in November [2020].” (1) Moreover, “an extraordinary number of COVID-19 trials have been registered since the pandemic started. The National Library of Medicine registry ClinicalTrials.gov lists 1087 COVID-19 studies…” (2).

Not only was the volume of information growing rapidly, but the speed at which new information was appearing unlike anything seen before. “Preprint postings in MedRxiv have increased over 400% (from 586 for the last 15 weeks of 2019 to 2572 for the first 15 weeks of 2020), while views and downloads have increased 100-fold.”(3)

It soon became difficult, if not impossible to separate the important from the mundane, the original from the repetition, and – most worryingly – the true from the false. Indeed, the miscommunications aspects of the pandemic soon began to appear like extended symptoms of the disease the communications were describing.

As a contribution to an understanding of the broader role of information in health development, this paper describes the principal antecedents in this new field, outlines its components, and considers some possible solutions and next steps.

## DEFINITIONS

Health information met the Internet long before the term “infodemiology” appears in the literature (4-6).Altogether, PubMed contains 16 314 mentions of the “World Wide Web” or “Internet” in biomedical literature before December 2002. Of these, 7 121 also used the term “information”, and 34 the term “epidemic”. So there had been numerous internet-based applications to both health and information before this research activity was named.

Prof. Gunther Eysenbach is credited as having coined the term "infodemiology" in 2002 (7). His original definition was “the study of the determinants and distribution of health information and misinformation—which may be useful in guiding health professionals and patients to quality health information on the Internet. Information epidemiology, or infodemiology, identifies areas where there is a knowledge translation gap between best evidence (what some experts know) and practice (what most people do), as well as markers for ‘high-quality’ information.” Later, he modified this definition to “the science of distribution and determinants of information in an electronic medium, specifically the Internet, with the ultimate aim to inform public health and public policy.” (8)

Both of Eysenbach’s definitions suggest information could be studied like a disease, that a cadre of infodemiologists would arise to supplement the epidemiologists. Since most epidemiologists are medical statisticians by training and spend their time gathering and analysing statistics, the implications of the name was that this kind of academic approach could be applied to infodemiology.

In a further paper (9), Eysenbach spells out the scope of the new field of infodemiology in more detail and offers a framework for it, introducing some basic metrics and distinguishing supply-side features (e.g., publishing activity on the Internet) from demand-based features (e.g., search and navigation behaviour). In general, he considered that “these metrics and methods are potentially useful for public health practice and research, and should be further developed and standardized.”

In this paper (9), Eysenbach also coined the terms “infoveillance” (for the systematic surveillance of information applications in public health) and “infodemic”, which he defined as “an excessive amount of unfiltered information concerning a problem such that the solution is made more difficult.”.

The new term was largely unused and forgotten until we reached the era of COVID-19, when Dr. Tedros Adhanom Ghebreyesus, the World Health Organization (WHO)’s Director-General, grappling with an epidemic, and later a pandemic, declared, at the Munich Safety Conference on February 15, 2020 (10), “We’re not just fighting an epidemic; we’re fighting an infodemic.”.

The shift of source metaphor (from epidemiology to epidemic) has also shifted the emphasis. According to WHO’s definition (11), “An infodemic is an overabundance of information – some accurate and some not – occurring during an epidemic. […] Like pathogens in epidemics, misinformation spreads further and faster and adds complexity to health emergency response.” Although Eysenbach’s definition of infodemiology suggested the field would engage in “the study of the determinants and distribution of health information and misinformation”, the redoubled emphasis on bad information (“some accurate and some not”) by WHO’s Director-General points the main focus of the field in that direction.

Epidemiology studies what is happening, while an epidemic *is* what is happening – one studies diseases, the other is itself a disease outbreak. This distinction is also true of infodemiology/infodemic. Both epidemiology and infodemiology nestle in the ivory towers of academe, generating papers and theories, while both epidemics and infodemics are events that require combat in the real world of public health.

Infodemic management is the practice of managing these information events with the aim of ensuring that everyone has access to the right health expert advice at the right time to be able to take appropriate action. This entails social listening (“infoveillance”) throughout a communication ecosystem and stakeholder engagement both online and offline. Because this is an emerging field, it will continue to evolve and possibly quite rapidly, especially given current “selection pressures”.

The field addressed by infodemiology has changed (and expanded) significantly over the last two decades. Rather than attempt a description of the field, however, here we will focus on an analysis of the characteristics of an infodemic.

## CHARACTERISTICS OF AN INFODEMIC

Using the WHO definitions, we can reduce the primary characteristics of an infodemic to 1) the volume of information generated, and 2) the velocity with which it appears. The most significant of the secondary characteristics of an infodemic are various forms of wrong information.

It may be asked if volume and velocity are both necessary and sufficient conditions for an infodemic; for example, if a large volume of largely true and helpful information about a health condition emerges quickly, would we still consider it an “infodemic”?

An answer could be that, from the perspective of an information manager, any large-scale and fast-flowing outburst of information needs to be managed. Information management includes sorting and classification, reviewing and judging for veracity and usefulness, translating and adapting knowledge to make it appropriate for different audiences, drawing conclusions and extracting recommended actions for decision-makers. It can often take a long time to identify whether any given information is in fact “helpful or true”, so the problems of volume and velocity remain for good and bad information alike.

The experience of the COVID-19 pandemic shows we are not yet equipped to cope with an infodemic, although the development of promising methodologies and software tools, including those based on artificial intelligence, is progressing rapidly. Table 1 sets out some of the primary characteristics of an infodemic.

The sudden increase in the volume of information experienced in an infodemic has immediate consequences on its location, quality, visibility, validity and capacity, while the speed at which new information is generated poses problems regarding assessment, gatekeeping, application and the ability to keep track of what we know. Both lead to poor coordination of the necessary research effort.

### Volume issues

As an infodemic starts, information begins to pour out from sources distributed geographically throughout the world. It emerges from conferences and meetings, leaks out of webinars and social media, makes use of all media, and shakes every grapevine in the community. Researchers struggle to identify, locate and collect it. This problem is greatly exacerbated by the digital divide, particularly in developing countries.

**TABLE 1.** Primary characteristics of an infodemic

Characteristic	Component	Effect
Volume of information	Location	Hard to find since it is scattered everywhere
	Capacity	Hard to collect, store and publish since there is so much of it
	Quality	Hard to identify the highest quality (as opposed to average or poor quality)
	Visibility	Hard for valuable new evidence to be seen/make an impact
	Validity	Hard to detect deliberate false news/lies/disinformation
Velocity of information	Assessment	Little time to weigh/analyse/judge it
	Gatekeeping	Little time to detect/refute mis/disinformation/rumours/lies
	Application	Delays in identifying/acting on correct information
	History	Hard to identify and keep track of the sequence of information events
	Waste	Research and results are unnecessarily replicated

Paradoxically, because it is so widely scattered, it becomes localised. People – whether information professionals or casual viewers – typically pick up their learning from a small selection of sources known to them, sources which they trust or believe. This is the “long tail” of the Internet at work (12). Thus, despite the global reach of the Internet, a curiously localised perspective emerges, since nobody can embrace the full volume of what is being generated. Nobody can collect, store and publish all of it. Consequently disagreements are plentiful.

With information overload, even dedicated information professionals and knowledge intermediaries like academic researchers and librarians find it hard to assess and rank the quality of what is emerging. This is a problem at the best of times: how sure can we be that even the research evidence published in the most respectable journals is accurate?

Most journals use peer review – the collegial assessment of manuscripts by peers (presumed equals to the authors) prior to publication as a way of identifying weaknesses and weeding out errors. It sounds like a good approach, but it has its critics.

One is Richard Smith, former editor of the *British Medical Journal*, who wrote (13), “Peer review is faith- not evidence-based, but most scientists believe in it as some people believe in the Loch Ness monster. Research into peer review has mostly failed to show benefit but has shown a substantial downside (slow, expensive, largely a lottery, wasteful of scientific time, fails to detect most errors, rejects the truly original, and doesn’t guard against fraud).”

So identifying what is right and wrong when the infodemic flood is at full spate is even more difficult than usual. As we know very well in science, it can take years before errors are recognized and corrected – Ptolemy was wrong in his astronomical theories, but it took 1300 years before Copernicus was able to confirm that the earth revolved around the sun, not the other way round. Or – to use a more recent biomedical example – Andrew Wakefield’s anti-MMR-vaccine paper was published in *The Lancet* in 1998, and was only retracted by the journal in 2010.

In the flood of information on the novel coronavirus, who can truly say what is 100% accurate? Oddly enough, it is often easier to detect straightforward mis/disinformation, than it is to guarantee the accuracy of genuine scientific information. Consider the shifting discussion on the efficacy of hydroxychloroquine in the early days of the coronavirus pandemic: the original research was published in what looked like an academic journal, so the information appeared to be valid; then the journal was accused of being a “predatory journal” (reducing the credibility of the information); and then the research was shown to be invalid in later research. And the debate about hydroxychloroquine continues (14).

We simply have to accept that some of what we consider to be valid information will ultimately prove to be false. Nevertheless, quality assessment is vital in an infodemic. We need more and better tools for this.

Other issues related to the volume of information in an infodemic include the difficulty for high-quality new information to gain visibility in the tsunami of mediocre commentary, and the concomitant ease for poor quality or erroneous information to survive unchallenged. Not to forget research in lower and middle income countries, which has always had a hard time gaining visibility: in an infodemic it simply goes unseen (15). In the avalanche of information, we could be missing much.

### Velocity issues

In an infodemic, with facts and factoids being posted at breakneck speed by countless sources, there is scant time to assess the validity or quality of new evidence. Society has so far not established the methods, knowledge management approaches and the cadre of dedicated and trusted specialists needed to carry out such assessments. This is true of intended truths, and it is all the more true of deliberate lies. We have to be able to identify worthwhile information quickly – and, equally, to discard the irrelevant and wrong as fast as possible. And yet, trusted gatekeepers capable of doing this simply don’t have the time to carry out the analysis.

The result of excessive velocity of information is that there are delays in applying correct information, and in suppressing incorrect information. A recent study published in the *American Journal of Tropical Medicine and Hygiene* (16) estimated that about 5 800 people were admitted to hospital as a result of false information on social media. The BBC reported that “at least 800 people died around the world because of coronavirus-related misinformation in the first three months of this year” (17). The article explains further, “Many died from drinking methanol or alcohol-based cleaning products. They wrongly believed the products to be a cure for the virus.” A quicker authoritative response to this idea could have saved lives.

Finally, with events moving so quickly in an infodemic, the true historical record becomes difficult to establish, making it easier for special interests to invent non-existent causalities. Were governments fast and agile enough when they should have been? Are we judging performance fairly according to the situation obtaining at the time, or are we indulging in hindsight? In which species and in which country did the virus originate?

The uncertainty and ambiguity caused by the new and unexpected lead to the need to consider multiple narratives at any given time, each of them potentially valid. For example, reactions to the virus outbreak were characterised by Nick Chater in *Nature* as “a storm in a teacup”, “a house on fire”, and “holding back the tide” (18). There was a massive effort in different countries to manage knowledge at the beginning of this epidemic – mainly to suppress or dismiss it as inconsequential (“storm in a teacup”). Then, as the scale of the problem grew uncontrollably, control measures were imposed – either radical (“house on fire”) or mitigating (“holding back the tide”). The ability to manage knowledge about the pandemic soon became uncontrollable. The alternative narratives highlight the importance of “multiple knowledges and multi-stakeholder processes in the solution of ‘wicked’ problems” (19).

Ultimately, the volume and velocity of information are by-products of the explosive nature of emergency information, whether good or bad. The problems of managing the velocity and volume of information are largely technical. Artificial intelligence and other technologies are likely to respond to the increased demands for quick and meaningful quality scanning and sorting.

### Misinformation, disinformation and malinformation

After volume and velocity, the third recognized characteristic of an infodemic is the spread of bad information – “bad” because it is simply wrong or useless (“misinformation”), or because in addition it has been deliberately twisted to accord with a political, ideological or other doctrinaire position (“disinformation”).

While WHO began to use the term “infodemic”, the United Nations Educational, Scientific and Cultural Organization (UNESCO) coined the term “disinfodemic” to focus on the bad information part of an infodemic (20). The Council of Europe identified yet another category of wrong information, namely “malinformation”, or “information that is based on reality, used to inflict harm on a person, social group, organisation or country” (21). In other words, malinformation is a form of disinformation produced maliciously, just to hurt people, rather than to further some specific ideological or political purpose. It is important to distinguish messages that are true from those that are false, but also those that are true, but which are created, produced or distributed by “agents” who intend to harm rather than serve the public interest. As such, malinformation is a cousin of “malware”, software designed to cause the user grief.

Concerns about misinformation hail back to the origins of the world wide web and even pre-date it (since misinformation flows through analogue communication systems equally well). Essentially, misinformation, disinformation and malinformation are different ways of getting it wrong. The first is an accident, while the other two are intentional. Of course, these categories do not remain distinct. People who pick up *dis*information and then pass it on simply because they believe it to be true (rather than to promote a particular political view or ideology) would then be spreading *mis*information – since they are sharing information that they honestly believe is true, but that someone else fabricated for their own reasons.

It appears that the experience of the pandemic has strengthened WHO’s focus on the disinformation part of the problem. “We’re not just battling the virus,” said WHO Director-General Tedros Adhanom Ghebreyesus. “We’re also battling the trolls and conspiracy theorists that push misinformation and undermine the outbreak response.” (22)

Finally, to complete this summary of bad information, there is another field in information management which has new relevance – namely agnotology. Agnatology is the study of ignorance. The term was suggested in 1995 by Proctor and Boal (23). As these authors write, “ignorance is often more than just an absence of knowledge; it can also be the outcome of cultural and political struggles. Ignorance has a history and a political geography, but there are also things people don't want you to know.” Thus, it is not enough to promote scientific literacy; ignorance has to be combated as well.

It is important to maintain the distinction between misinformation and disinformation, because the solutions to each are likely to be different. Large doses of good information will certainly help in the struggle against misinformation, as will a renewed effort on improving scientific literacy. But disinformation will use the same channels as good information and do everything possible to be indistinguishable from it.

The urge to issue disinformation has psychological, socio-political and cultural roots. For a variety of reasons, people are deliberately poisoning the well. The solutions to this problem can only come from society. For example, there are arguments for invoking anti-trust laws to break up the big data/social media concerns – which often act as global monopolies – into smaller, more-manageable pieces. Others advocate using legal restrictions as the best cure for disinformation – some form of punitive action, fines, public scorn, prohibitions on publication in social media and elsewhere, and the like. It may be time to make Internet publishers (mainly the social media platforms) liable for the content they publish, just as publishers in the analogue world face legal consequences if they promote incitements to violence, abuses of human rights, the proliferation of hate and other illegal speech, and the like.

Given the non-national nature of the Internet, many of these actions would require an international legal framework. Such frameworks typically take a long time to agree – if they are ever agreed at all.

Moreover, over-arching all regulatory and technical issues, major human rights concerns need to be addressed, to ensure that remedial actions do not lead to censorship and the curtailment of free speech.

Technology alone will not provide a solution. Unfortunately, any new mechanisms used to spread good information can also be used to share false and harmful messages. Artificial intelligence can work both ways. So far, technological progress has not improved this situation. It has often made it worse by offering new outlets, and new ways of bending the truth. Digital innovations that can help shift the way that technology and social media are currently used are needed. There’s an opportunity for the sector to take a leadership role in driving solutions. Another proposal that has been aired repeatedly since the birth of the Internet is to establish a safe space for trusted information – a top-level internet domain that will house websites which have been vetted and approved for this purpose. This is discussed in the next section.

## CREATING A TOP-LEVEL DOMAIN FOR TRUSTWORTHY HEALTH INFORMATION^1^

The idea of setting up a trusted top-level domain (TLD) for health information has been around since the birth of the world wide web. In 1998, the World Health Organization began working on a proposal to establish a top-level domain called .health. This was to provide a safe home for websites that had been certified as being trustworthy by one of the kite-marking schemes that appeared in the late 1990s.

One of the longest-established of these kitemarkers is Health On the Net (25), which applies an ethical code in providing a certificate to websites dealing with health information. The HON Code is based on eight principles: a site can be certified if it is authoritative, supports (not replaces) the relationship that exists between patient and physician, respects privacy, provides attribution to source data, justifies opinions with evidence, is transparent, discloses its financial backing, and has a defensible advertising policy.

HON claims it has certified over 8 000 websites in the quarter of a century since 1995. While this is impressive, it remains a small number, when one considers that, as reported in one article, “…more than 1 in 10 news websites accessed by Americans includes bad information about health” (26). An individual cited in that article was reported to have created over 200 websites promoting anti-vaccine disinformation. When one considers what one determined person can do, it is likely that a myriad sites on the Internet are actively disseminating disinformation. HON has a way to go.

Still, WHO started to lobby for a new .health domain. On 2 October 2000, WHO submitted a formal application to the Internet Corporation for Assigned Names and Numbers (ICANN) to create and manage (through the CORE registrar, a subcontractor) a .health domain: “WHO proposes .health as a restricted TLD dedicated to screened health information providers, as distinguished from the unregulated information on general TLDs.” (18)

ICANN initially evaluated this positively, noting that “The strengths of the application lie in the WHO’s international influence in the health community, the value of increased access to trusted health information, and CORE’s registrar experience… Overall, this application could lead to a successful new TLD given its limited objectives, the technical background of the operator and the altruistic purposes of the TLD.”

Public commentary in support of the application maintained that WHO would “provide neutral, international support for standards of health information on the Internet”, that it could provide quality control, and even that “establishing a .health TLD would greatly enhance, if not revolutionize health and medical electronic communication, telemedicine, medical care in both industrialized and developing countries, medical and health technology transfer as well as having a DIRECT impact on reducing and preventing disease and disability and thus reducing overall human suffering.”

However, objectors suggested that “.health is too narrow and may not have any real value to the general e-public”, “if WHO wished to vet websites, it could do it through its own website, as opposed to controlling an entire TLD”, “can WHO truly exercise independent judgment given that it relies upon the good graces of national governments to operate in many parts of the world?” Some commentators worried that vetting would likely be an expensive proposition – how would WHO fund these operations? The proposal was turned down.

Still, the idea did not go away. In 2013, the Member States of WHO’s governing body, the World Health Assembly declared (27): “…health-related global top-level domain names in all languages, including “.health”, should be operated in a way that protects public health, including by preventing the further development of illicit markets of medicines, medical devices and unauthorized health products and services, and urged the WHO Director-General “…to convey to the appropriate bodies, including the ICANN Governmental Advisory Committee and ICANN constituencies, the need for health-related global top-level domain names in all languages, including “.health”, to be consistent with global public health objectives.”

Accordingly, WHO made a second concerted attempt to manage a .health TLD and submitted another formal application to do so. Despite intense lobbying by WHO and its supporters, and interventions by governments, once again, it was unsuccessful. Commercial considerations appeared to prevail. The domain eventually went to the dotHealth company, and it has been operated as a commercial enterprise since 2017 (28).

Although the dotHealth submission to ICANN contained “policy commitments” that included “explicit prohibitions against the use of .health domain names for illicit drugs, abusive commercial practices targeted at consumers and children, and for pornographic materials depicting individuals under the age of majority in the relevant jurisdiction” – there has been no evident gatekeeping regarding the quality or accuracy of the information published on the domain (29). This concept is certainly not actively promoted on the dotHealth website.

Looking at it from the perspective of the COVID-19 infodemic, and notwithstanding WHO’s discouraging experiences with .health to date, it really might be a good moment to try again. A new top-level domain could be established to provide a sheltered space for health information without ideological or political bias. Such a domain could be designed as a space analogous to international waters, and managed by an independent international partnership that would provide the independent monitoring of content and establishment of new processes for health sites to meet internationally agreed standards.

## DISCUSSION

The critical role of information – and knowledge – in well-functioning societies has been driven home emphatically by the COVID-19 infodemic, and new work in this area is finding widespread interest. It is time to integrate this into the wider development effort.

The framework for the current international development agenda is outlined in the United Nations’ Sustainable Development Goals (SDGs)(30). The SDGs cover most areas of economic and social development. Each of the Goals has specific targets and associated indicators, which are used as direct planning tools, particularly in the health sector (which falls mainly under SDG3).

Unfortunately, there is no SDG for information or knowledge management, as these were assumed to be cross-cutting elements affecting all of the Goals. While it may be true that information and knowledge are intrinsic to all human endeavour, the absence of a specific Goal about improving access to and use of essential information, and the management of knowledge, hinders the formulation of the kind of concerted efforts that will be required to address the problems of an infodemic. Without a Goal, it is difficult to formulate internationally agreed targets and indicators, and next to impossible to secure significant development funding for such work. At present, the Goal relating to health does not mention the role of essential health information – there is surely an opportunity to change this in the wake of the COVID-19 infodemic.

The SDGs framework as part of Agenda 2030 will need to be revised or replaced in the coming years so there will be an opportunity to build health information management into that framework. Specifically, it may be possible to create a new indicator for SDG3 related to access to reliable health information as a human right to enable informed decision-making about personal and public health. Ultimately, the right to health necessarily entails access to the information needed to make decisions for protecting health.

Finding useful indicators to measure information aspects has always been a problem, and this is where the new field of infodemiology can help. New approaches to knowledge management have also been spurred by the infodemic. In July 2020, the Knowledge Management for Development group conducted a lengthy discussion on “Coronavirus & KM”, concluding that a new approach was needed, and offering a “KM Preparedness Strategy: Knowledge Management for Epidemics/ Pandemics” (31).

In April 2020, WHO organized a two-day global consultation to “crowdsource ideas to form a novel COVID-19 infodemic response framework”. Some 50 actions were suggested for the framework as well as six key implications for governments and policy makers to consider – these are described in the published report (32). They are a first attempt, and will need elaboration as new ideas arise.

WHO’s repeated attempts at establishing a .health top-level domain on the internet under its management illustrate the difficulties of bringing what are seen as ethical and “altruistic” concerns into a field dominated by commercial considerations. Although such a development would be good ethics, there would be nothing altruistic about it, since trusted information has a value, as we have seen repeatedly during the COVID-19 pandemic. Such a top-level domain would fulfil the objective of supporting universal health coverage by providing a robust and trusted receptacle for essential healthcare information. This is needed if the Sustainable Development Goals agreed by all countries are to be achieved. Good health leads to better livelihoods, and global health should lead to global prosperity. The creation of a new trusted top-level domain would require a very broad consortial approach, with a multi-stakeholder platform that includes a range of stakeholder groups as part of a broader social movement. It could be multisectoral (not just focusing on health). There would have to be a very tight and well-organized process for admitting members and websites and information, and it would have to be curated to the highest standards. International standard setting (and community norm setting) would be needed.

## CONCLUSIONS

This paper has traced the recent evolution of the concept of infodemiology from its original academic beginnings as a form of epidemiology mainly applied to information on the Internet, to the applied version of an infodemic accompanying a pandemic. It has described the characteristics of an infodemic, which combines a diseased output of information, in terms of both volume and velocity, as well as a collateral growth of misinformation and disinformation.

The solutions proposed include 1) a new trusted top-level domain for health information, which will also spur the development of standards and bring together a consortium of stakeholders; 2) a new SDG target and goal on health information to focus activities towards combating infodemics arising in future; and 3) knowledge preparedness plans that are appropriate to every country’s information culture and needs. Other solutions to the problems arising will be technical, social and economic, but they will also have to be approached through a human rights lens. COVID-19 has certainly changed the world, causing much suffering; it has also established the imperative for accelerated progress in infodemic management. The results of that work will impact on the way we manage future public health emergencies and, more generally, on the way we communicate as a species.

## Disclaimer.

Author holds sole responsibility for the views expressed in the manuscript, which may not necessarily reflect the opinion or policy of the RPSP/PAJPH and/or PAHO.

## Footnotes

1 All source information regarding the WHO applications for a .health domain is from the ICANN archive (24)
